# Intramelanocytic Acidification Plays a Role in the Antimelanogenic and Antioxidative Properties of Vitamin C and Its Derivatives

**DOI:** 10.1155/2019/2084805

**Published:** 2019-05-12

**Authors:** Fang Miao, Meng-Yun Su, Shan Jiang, Long-Fei Luo, Ying Shi, Tie-Chi Lei

**Affiliations:** Department of Dermatology, Renmin Hospital of Wuhan University, Wuhan 430060, China

## Abstract

Although vitamin C (VC, L-ascorbic acid) has been widely used as a skin lightening agent for a long time, the mechanism by which it inhibits melanogenesis remains poorly understood. It is well-documented that the intramelanocytic pH is an important factor in regulating tyrosinase function and melanosome maturation. The activity of tyrosinase, the rate-limiting enzyme required for melanin synthesis, is generally minimal in an acidic environment. Given that VC is an acidic compound, we might speculate that the intracellular acidification of melanocytes induced by VC likely reduces melanin content through the suppression of tyrosinase activity. The results of this study reveal that treatment of melanocytes with VC or its derivatives, magnesium ascorbyl phosphate (MAP) and 3-O-ethyl-L-ascorbic acid (AAE), resulted in significant decreases in the tyrosinase activity and melanin content and in the levels of intracellular reactive oxygen species (ROS), indicating that VC and its derivatives possess antimelanogenic and antioxidative activities. Western blotting analysis indicated that VC, MAP, and AAE exert their antimelanogenic activity by inhibiting the tyrosinase activity rather than by downregulating the expression of melanogenic proteins such as tyrosinase, premelanosome protein 17 (Pmel17) and microphthalmia-associated transcription factor (MITF). Further, we found that the reduced tyrosinase activity of melanocytes treated with VC or its derivatives could be reactivated following intracellular neutralization induced by ammonium chloride (NH_4_Cl) or concanamycin A (Con A). Finally, we examined the expression of sodium-dependent VC transporter-2 (SVCT-2) using western blotting and qPCR, which revealed that there was a significant increase in the expression of SVCT-2 in melanocytes following treatment with VC. VC-mediated intracellular acidification was neutralized by phloretin (a putative SVCT-2 inhibitor) in a dose-dependent manner. Taken together, these data show that VC and its derivatives suppress tyrosinase activity through cytoplasmic acidification that potentially results from enhanced VC transmembrane transport via the VC transporter SVCT-2.

## 1. Introduction

Melasma (chloasma) is a common skin pigmentary disorder characterized by irregular light- to dark-brown patches on the face, which usually cause significantly psychiatric and psychological burdens for affected individuals. Although many options such as laser and light therapies are available for subjects with refractory melasma, those therapies carry a significant risk of worsening the disease in some individuals. Hydroquinone (HQ) represents a prototypic medication that is used to treat melasma; however, many biosafety concerns have been raised in recent years with regard to the long-time use of HQ as an active ingredient supplemented in cosmetic products and daily necessities. There are also several clinical reports showing that exogenous ochronosis and irreversible skin depigmentation can potentially occur in individuals who are exposed to large doses of HQ over extended time periods. Therefore, a safe and effective alternative to HQ for use in skin lightening is highly desirable [1].

Vitamin C (VC), also known as ascorbic acid, is a water-soluble vitamin essential for a number of processes in human skin, such as dermal collagen synthesis, antiaging, and antioxidation [2–4]. Emerging evidence has indicated that VC and its derivatives exert therapeutic effects on recalcitrant melasma and facial hyperpigmentation [5, 6], but little is known about their antipigmentary mechanism(s). VC is a weak acid (pKa = 4.2) and is only slightly stronger than vinegar [7]; thus, we assume that the acidification of melanocytes (MCs) by VC could inhibit the catalytic activity of tyrosinase, the rate-limiting enzyme required for melanin biosynthesis, through increased transmembrane transport of VC. This study was designed to determine (i) whether the intracellular pH of MCs is changed following treatment with VC or its derivatives, (ii) whether the reduced tyrosinase activity can be restored by neutralizing the intracellular pH, (iii) whether VC has a scavenging effect against reactive oxygen species (ROS) in MCs following UVA-mediated oxidative stress, and (iv) whether enhanced transmembrane transport of VC is involved in the expression levels of sodium-dependent vitamin C transporter isoforms (SVCT-1 or/and SVCT-2). In addition, premelanosome protein 17 (Pmel17) is a pH-sensitive protein that forms the hierarchically assembled amyloid fibrils required for melanin deposition [8]; microphthalmia-associated transcription factor (MITF) functions as a master regulator of melanosome maturation and function [9]; therefore, we also examined the changes of MITF and Pmel17 proteins in the MCs upon acidification induced by VC.

## 2. Materials and Methods

### 2.1. Cell Culture and Treatment

**Figure 1:**
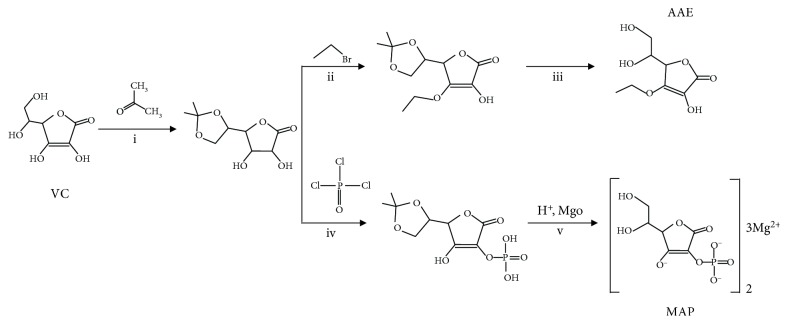
Synthetic route of MAP and AAE. (i) The first step in the synthesis of both compounds was protection of the 5,6-position hydroxy of VC. VC was reacted with three equivalents of acetone to form 5,6-O-isopropylidene-L-VC under catalysis of 0.1 equivalent of Lewis acid. (ii) 5,6-O-isopropylidene-L-VC was reacted with 1.1 equivalents of ethyl bromide to form the 3-O-ethylated intermediate. (iii) The 3-O-ethylated intermediate was hydrolyzed with 0.1 equivalent mineral acid to form AAE. (iv) 5,6-O-isopropylidene-L-VC was reacted with phosphorus oxychloride to form the phosphate intermediate under alkaline conditions. (v) The phosphate intermediate was deprotected by acid hydrolysis to form VC-2-phosphate, after which it was reacted with magnesium oxide to form MAP.

Melan-a MCs, an immortalized and nontumorigenic line of MCs derived from C57BL/6J (black, a/a) mice, were maintained in complete RPMI 1640 medium supplemented with 10% (*v*/*v*) fetal calf serum, 1% (*v*/*v*) penicillin/streptomycin (100 units/ml), 100 *μ*M 2-mercaptoethanol, 2 mM L-glutamine, and 200 nM phorbol myristate acetate at 37°C in a humidified atmosphere with 5% CO_2_. Primary human MCs were isolated from juvenile foreskin tissues and were cultured with complete Medium 254 (Gibco, Invitrogen, Carlsbad, CA, USA) supplemented with human melanocyte growth supplement (Gibco). Concanamycin A (Con A, a selective vacuolar-type H^+^-ATPase inhibitor), *α*-melanocyte-stimulating hormone (*α*-MSH), HQ, and VC were purchased from Sigma-Aldrich Corp. (St. Louis, MO, USA). Ammonium chloride (NH_4_Cl) was obtained from BBI Life Sciences (Shanghai, China). Phloretin (a putative SVCT-2 inhibitor) was purchased from Selleck Chemicals (Cat# S2342, Shanghai, China) [10, 11]. Magnesium ascorbyl phosphate (MAP) and 3-O-ethyl-L-ascorbic acid (AAE) were synthesized in our laboratory according to the synthetic route shown in Figure 1. VC, MAP, and AAE were dissolved in phosphate buffer solution (PBS, pH = 7.0) and added into fresh cell culture medium at the indicated concentrations. The cell culture medium containing VC, MAP, or AAE was always buffered to pH 7.0 and was prepared immediately before use. For neutralization, the MCs were treated with VC, MAP, or AAE for 24 h and then were additionally treated with 10 nM Con A or 10 mM NH_4_Cl for another 24 h. The details of the methods are indicated in the figure legends.

### 2.2. Cell Viability Assay

Cell counting kit-8 (CCK-8) reagent (Beyotime Biotechnology, Nanjing, China) was used to determine cell viability according to the manufacturer's instructions. In short, MCs were seeded at 5 × 10^3^ per well in 96-well plates, allowing the cells to attach to the bottom of the wells. The MCs were then treated with various concentrations of VC, MAP, or AAE. After 48 h, 20 *μ*l CCK-8 was added to each well and incubated at 37°C for 1 h. The optical density (OD) value was recorded at 450 nm using a microplate reader (PerkinElmer, Waltham, MA, USA).

### 2.3. Melanin Content Assay

Normal human MCs were seeded into 6-well plates for 24 h and then fed fresh medium containing 2 *μ*M *α*-MSH, 3 *μ*M HQ, 1 mM VC, 1 mM MAP, or 1 mM AAE. PBS served as the control. After 48 h, the MCs were centrifuged (1000 g, 5 min) and cell pellets were photographed using a digital camera. Subsequently, the harvested MCs were washed twice with PBS and were then suspended in 100 *μ*l 1 mM NaOH and heated at 80°C for 1 h to dissolve melanin. The absorbance at 405 nm was read using a microplate reader. The results are presented as the fold of the treated group compared with the control.

### 2.4. Tyrosinase Activity Assay

Tyrosinase activity was determined using a previously published protocol [12]. Briefly, MCs were harvested and solubilized in extraction buffer (pH 7.2) containing 1% Nonidet P-40, 0.01% sodium dodecyl sulfate (SDS), and protease inhibitor cocktail (Roche, Indianapolis, IN, USA). Protein contents were quantified using a BCA assay kit (Beyotime Biotechnology, Nanjing, China). The same amount of protein in each extract (≈10 *μ*g) was spotted on polyvinylidene difluoride (PVDF) membranes (Millipore, Billerica, MA, USA) using a dot blot apparatus (Bio-Rad, Hercules, CA, USA) and immediately incubated with L-3,4-dihydroxyphenylalanine (L-DOPA) at 37°C. The membranes were then washed twice with PBS and air-dried. Images of each PVDF membrane were taken using a digital camera, and the gray value of each dot was measured using ImageJ software (NIH, Bethesda, MD, USA).

### 2.5. Acridine Orange Fluorescent Staining

To visualize acidic organelles in MCs, the lysosomotropic weak base acridine orange (AO) (Sangon Biotech, Shanghai, China) was used for analysis. AO can be taken up by MCs and accumulates in acidified compartments such as lysosomes, endosomes, and melanosomes. The fluorescence intensity of AO is green at low concentrations and orange when it accumulates at high concentrations in acidic organelles. In brief, MCs were seeded on coverslips in 6-well plates and allowed to attach overnight. After treatment with various compounds for 48 h, the medium was replaced with fresh medium supplemented with AO (5 *μ*g/ml) and incubated for an additional 30 min. For examining the effect of SVCT-2 function on intracellular acidification, the cells were cotreated with VC and phloretin for 48 h. Cell nuclei were then stained with 50 *μ*l of a working solution of 4,6-diamidino-2-phenylindole (DAPI). The cells were examined and photographed using a FV1200 confocal imaging system (Olympus, Tokyo, Japan). The red fluorescence intensity of acridine orange (an indicator for acidic compartments) was analyzed using ImageJ software (NIH, Bethesda, MD, USA).

### 2.6. Acidic Organelle Staining with Fluorescent Probes

LysoSensor Green DND-189 (Invitrogen) is an acidotropic fluorescence probe which becomes fluorescent in acidic compartments, and its fluorescence intensity is highly correlated with intracellular pH [13]. After treatment with various compounds for 48 h, 1 *μ*M LysoSensor Green DND-189 was added into the cell medium and incubated for 30 min in the dark. After incubation, the MCs were washed and fluorescence images were acquired by confocal microscopy at a 488 nm excitation wavelength. The fluorescence intensity was measured using ImageJ software (NIH, Bethesda, MD, USA).

### 2.7. Western Blotting Analysis

The MCs were harvested and washed in PBS and then lysed in extraction buffer. Protein contents were determined using a BCA assay kit (Pierce, Rockford, IL, USA). Equal amounts of each protein extract (20 *μ*g per lane) were separated by 10% SDS polyacrylamide gel electrophoresis. Following transfer to PVDF membranes and blocking with 5% nonfat milk diluted in Tris-buffered saline, the membranes were incubated with antibodies to tyrosinase (Cat# bs-0819R, Bioss, Beijing, China), Pmel17 (Cat# ab137078, Abcam, Cambridge, MA, USA), microphthalmia-associated transcription factor (MITF) (Cat# ab20663, Abcam), SVCT-2 (Cat# PA5-42447, Invitrogen), and GAPDH (Santa Cruz Biotech, Santa Cruz, CA, USA) overnight at 4°C. GAPDH was used as loading control. The PVDF membranes were then washed with TBS with Tween-20 (TBS-T) and then were further incubated with horseradish peroxidase-conjugated secondary antibodies (Pierce) at a dilution of 1 : 2000 for 1 h at room temperature. Each membrane was then washed again, and protein bands were visualized by an enhanced chemiluminescence (ECL) detection system (Amersham, Piscataway, NJ, USA). The intensity of each band was quantified using ImageJ software and was normalized against GAPDH.

### 2.8. UVA Radiation and Intracellular ROS Assay

Intracellular ROS levels were measured as described previously [14]. MCs were seeded in 6-well plates at a density of 2 × 10^5^ cells/well. After 24 h, VC was added into the medium and the MCs were further incubated for an additional 24 h. The cells were exposed to 3 J/cm^2^ UVA radiation from a bank of nine Philips UVA lamps (320-380 nm) with a peak emission at 350 nm (SIGMA High-tech Co., Ltd., Shanghai, China) according to a published protocol with minor modification [15]. The dose of UVA irradiation was calibrated with a digital radiometer (SIGMA High-tech Co., Ltd., Shanghai, China) prior to each exposure. Cell culture plates were wrapped with aluminum foil for sham-irradiated controls. Finally, the MCs were loaded with 10 *μ*M 2,7-dichlorofluorescein diacetate (DCFH-DA) (Sangon Biotech) in serum-free medium at 37°C for 30 min in the dark. DCFH-DA-labeled cells were observed using a fluorescence microscope. For qualitative analysis, flow cytometry was used to measure the fluorescence intensity of cells. Approximately 20,000 MCs were collected for each sample using a BD FACSCalibur flow cytometer (BD, Franklin Lakes, NJ, USA) equipped with CellQuest Pro software.

### 2.9. Quantitative Real-Time Polymerase Chain Reaction (qPCR)

Total RNA was isolated from MCs using TRIzol reagent (Invitrogen), and first-strand complementary cDNA was synthesized using a PrimeScript RT reagent kit (TaKaRa Biotech, Beijing, China). qPCR was conducted using a SYBR Premix Ex Taq™ kit II (TaKaRa). SYBR green real-time PCR mix containing 10 *μ*M forward and reverse primers of the SVCT-2 gene (forward: 5′-CAT GCT GAC GAT TTT CCT AGT G-3′; reverse: 5′-TGA AAA GCT GGA ACT TGT ATG C-3′) and the SVCT-1 gene (forward: 5′-AGG TGC TAT CAA CAC AGG CAT T-3′; reverse: 5′-TAA TGT TGA AGG TCA AGC CCA GG-3′). Real-time PCR was performed using ABI 7500 and cycle parameters as follows: denaturation at 95°C for 10 min; 40 cycles of 95°C for 30 s, 55°C for 30 s, and 72°C for 30 s. The purity of each qPCR product was checked by its dissociation curve. Primers were purchased from Sangon Biotech and GAPDH was used as the reference gene. Relative quantification of gene expression levels for target and reference genes was performed by the 2^-△△Ct^ method and based on Ct values.

### 2.10. Statistical Analysis

All data are expressed as means ± standard deviation of at least three independent experiments. Statistical analyses were performed with SPSS version 19.0 (IBM SPSS, Armonk, NY, USA) and GraphPad Prism (San Diego, CA, USA) software. Comparisons were made using an unpaired two-tailed Student's *t* test. One-way analysis of variance (ANOVA) was used to evaluate differences between three or more groups followed by Tukey post hoc test. *P* < 0.05 is considered to be a statistically significant difference.

## 3. Results

### 3.1. The Antimelanogenic Effect of VC and Its Derivatives Is due to Suppressed Tyrosinase Activity rather than the Downregulated Expression of Melanogenic Proteins

**Figure 2:**
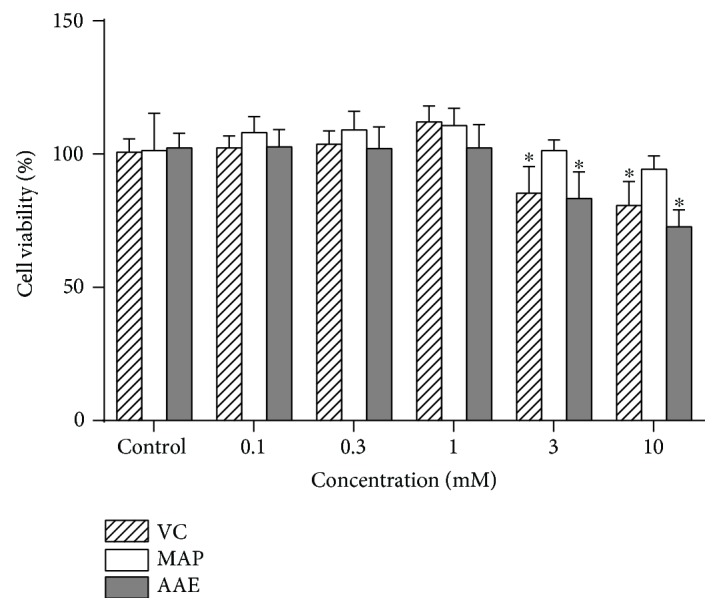
Cell viability of MCs exposed to VC, MAP, or AAE. MCs were seeded in 96-well plates and then were treated with varying concentrations (0, 0.1, 0.3, 1, 3, and 10 mM) of VC, MAP, or AAE for 48 h. Cell viability was determined using the CCK-8 assay as described in Materials and Methods. All data are presented as means ± SD for each treated group compared with the control group from three independent experiments. ^∗^ *P* < 0.05 versus the control group.

**Figure 3:**
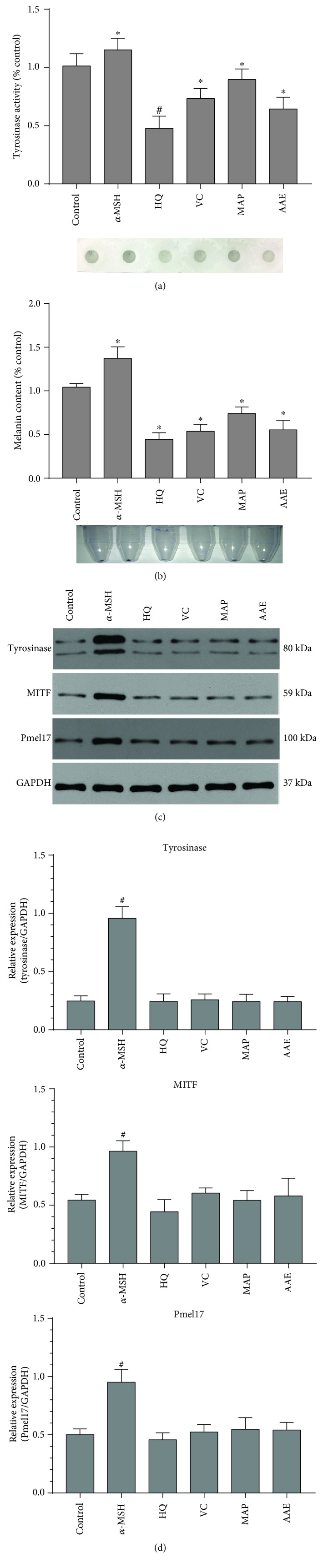
Effects of VC, MAP, or AAE on the tyrosinase activity, melanin content, and the expression level of melanogenic proteins. (a) MCs were treated with 1 mM VC, MAP, or AAE for 48 h, after which the tyrosinase activity of treated MCs was measured using the dot-blot assay as described in Materials and Methods. Results are given as the percentage compared to the control group. Representative dot images for the detection of tyrosinase activity immobilized on PVDF membranes are shown at the bottom. (b) Primary human MCs were treated with the indicated compounds for 48 h, and then, melanin content was measured using spectrophotometric analysis. Representative cell pellet images are shown at the bottom. (c) Treated or untreated human MCs were harvested, and equal amounts (10 *μ*g per lane) of each protein extract were resolved using 10% SDS-PAGE electrophoresis. Protein loading variations were determined by immunoblotting with an anti-GAPDH antibody. Representative blots are shown. (d) Histograms showing the densitometric quantification of data with means ± SD of three independent experiments. ^∗^ *P* < 0.05, ^#^ *P* < 0.01, compared to the untreated control.

In this study, we initially determined the tyrosinase activity, melanin content, and proliferation rates of MCs exposed to VC, MAP, or AAE or with *α*-MSH (a known melanogenic stimulator) or HQ (a known melanogenic inhibitor) as controls. When MCs were treated with VC, MAP, or AAE for 48 h, no discernible cytotoxic or cell growth inhibitory effects were observed at concentrations of 1 mM or lower (Figure 2). However, the tyrosinase activity and melanin content were significantly decreased in MCs treated with 1 mM VC, MAP, or AAE (Figures 3(a) and 3(b)). We also examined the expression levels of tyrosinase, MITF, and Pmel17 proteins in treated or untreated MCs using western blotting. There was no significant reduction in protein levels of tyrosinase, MITF, or Pmel17 in MCs treated with VC, MAP, or AAE compared with the untreated control (Figures 3(c) and 3(d)). As expected, more than a 3-fold increase in the level of tyrosinase protein was observed after treatment with 2 *μ*M *α*-MSH for 48 h. These results show that VC and its derivatives possess skin lightening properties due to the inhibition of tyrosinase activity and not by the decreased expression of melanogenic proteins.

### 3.2. The Antimelanogenic Effect of VC and Its Derivatives on MCs Is Abrogated by Neutralizing Intracellular pH

**Figure 4:**
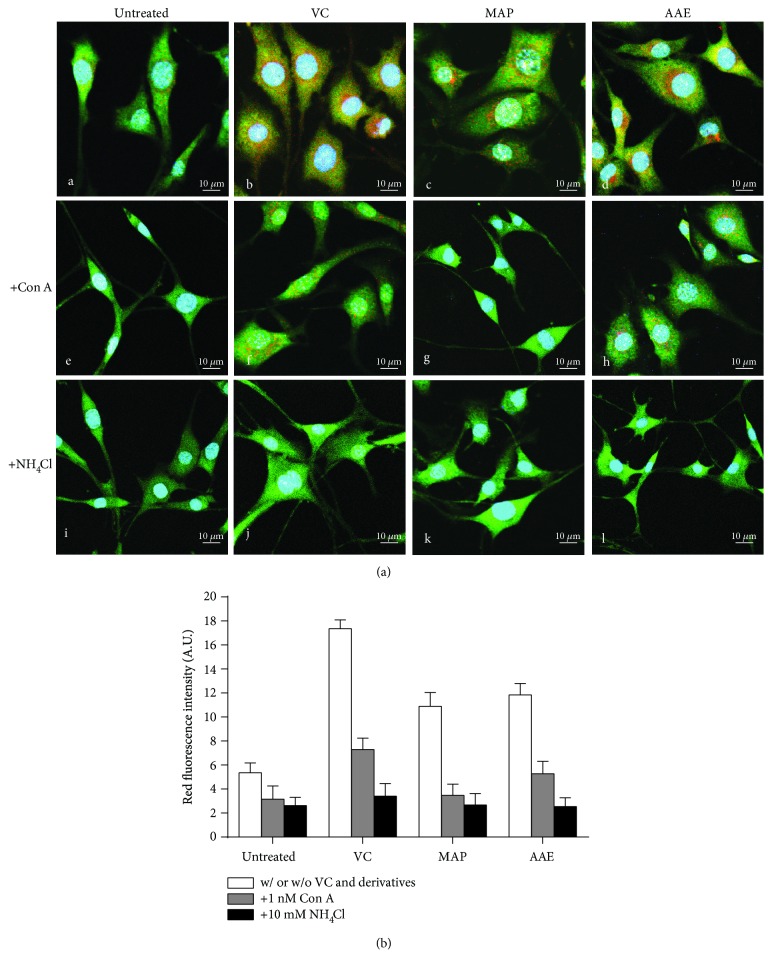
Visualization of cytoplasmic acidification in treated MCs using AO fluorescent staining. (a) MCs were seeded on coverslips in a 6-well plate and then untreated (A, E, and I) or treated with VC (B, F, and J), MAP (C, G, and K), or AAE (D, H, and L) for 48 h. After treatment, the MCs were incubated with fresh medium containing 5 *μ*g/ml AO for 30 min. The red fluorescence staining indicates the acidic cytoplasmic compartment. For pH neutralization, the MCs were simultaneously untreated (A–D) or treated with 10 nM Con A (E–H) or 10 mM NH_4_Cl (I–L). Nuclei were stained with DAPI (blue). Scale bar: 10 *μ*m. (b) Red fluorescence intensity (arbitrary units (A.U.)) for AO staining was measured using ImageJ. Histogram shows the results determined on 50 cells which are presented as means ± SD for three independent experiments. ^∗∗^ *P* < 0.01 versus only VC or its derivatives.

**Figure 5 fig5:**
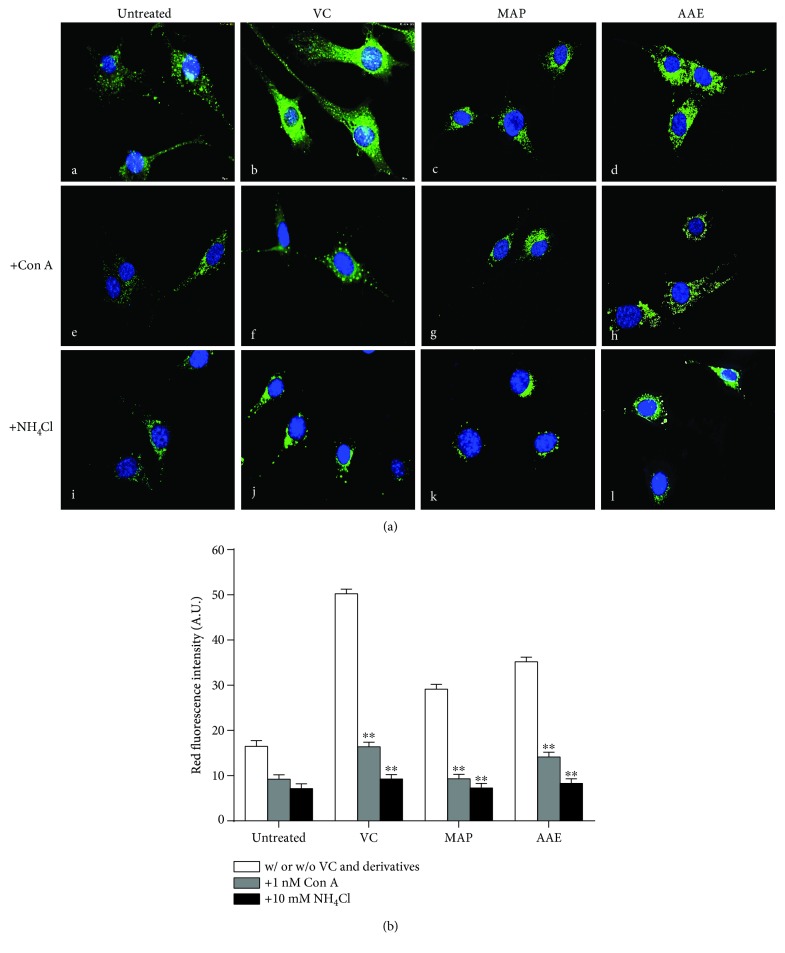
Change of cytoplasmic pH in treated MCs using LysoSensor Green DND-189 staining. (a) MCs were seeded on coverslips in a 6-well plate and then were untreated (A, E, and I) or treated with VC (B, F, and J), MAP (C, G, and K), or AAE (D, H, and L) for 48 h. After treatment, the MCs were incubated with fresh medium containing 1 *μ*M LysoSensor Green DND-189 for 30 min in the dark. The green fluorescence staining indicates the acidic cytoplasmic compartment. For pH neutralization, the MCs were simultaneously untreated (A–D) or treated with 10 nM Con A (E–H) or 10 mM NH_4_Cl (I–L). Nuclei were stained with DAPI (blue). Scale bar: 10 *μ*m. (b) Fluorescence intensity (arbitrary units (A.U.)) for LysoSensor Green DND-189 staining was measured using ImageJ. Histogram shows the results determined on 50 cells which are presented as means ± SD for three independent experiments. ^∗∗^ *P* < 0.01 versus only VC or its derivatives.

**Figure 6:**
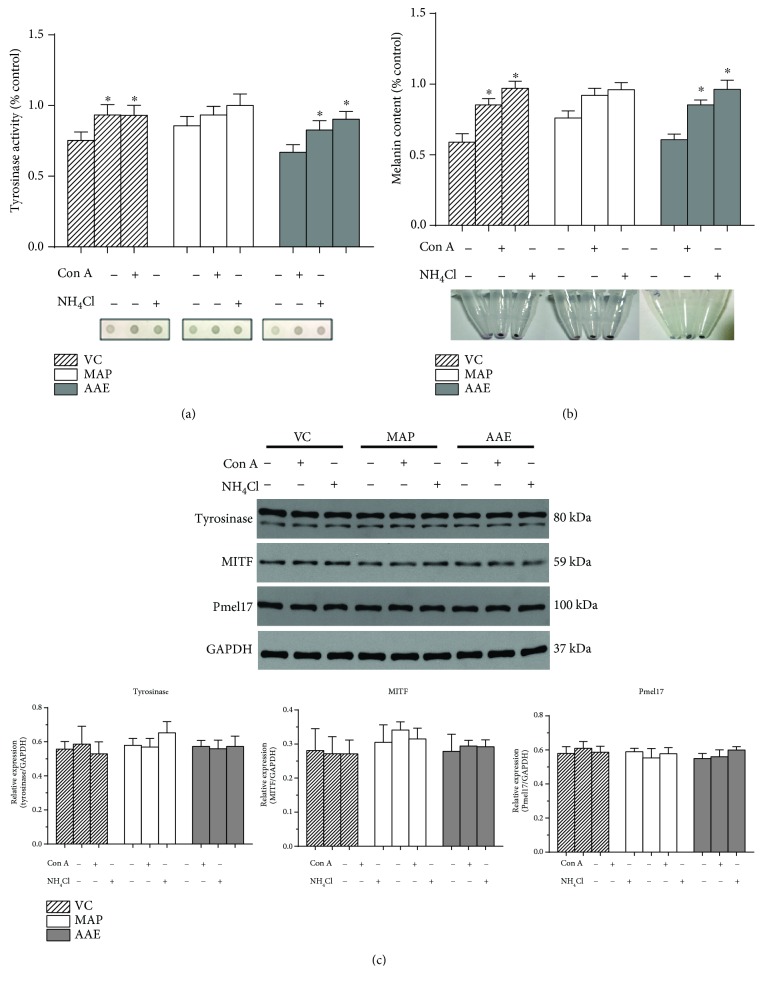
Effect of intracellular neutralization on the tyrosinase activity, melanin content, and expression level of melanogenic proteins. MCs were untreated or treated with 1 mM VC, MAP, or AAE for 48 h; the tyrosinase activity (a) and melanin content (b) of treated MCs were measured by dot-blot assay and spectrophotometric analysis, as detailed in Materials and Methods. For pH neutralization, the MCs were simultaneously untreated or treated with 10 nM Con A or 10 mM NH_4_Cl. (c) The expression levels of tyrosinase, MITF, and Pmel17 proteins were analyzed by western blotting using anti-tyrosinase, anti-MITF, and anti-Pmel17 antibodies. GAPDH was used as a loading control. Representative blots are shown. Data are shown as means ± SD of three independent experiments. ^∗^ *P* < 0.05 versus only VC or its derivatives.

First, we examined whether exposure to VC, MAP, or AAE would affect the intracellular pH of MCs using two pH-sensitive fluorescent dyes. AO is a cell-permeable basic dye that can be selectively accumulated in acidified compartments in living cells, such as lysosomes and melanosomes, and emits red-orange fluorescence. As shown in Figure 4, MCs loaded with AO in the presence of VC, MAP, or AAE showed an intense red-orange fluorescence. After intracellular neutralization of MCs induced by NH_4_Cl or Con A, the red-orange fluorescence became almost invisible. Similar results were obtained from other independent experiments using LysoSensor Green DND-189 staining. LysoSensor Green DND-189 is also a pH-sensitive dye that is brightly fluorescent upon acidification and can be used to measure the pH of acidic organelles. MCs loaded with LysoSensor Green DND-189 in the presence of VC, MAP, or AAE showed an intense green fluorescence, whereas that fluorescence was markedly diminished by exposure of those cells to Con A or NH_4_Cl (Figure 5). Next, we investigated whether the cytoplasmic neutralization of MCs reduced the antimelanogenic effects of VC and/or its derivatives (Figures 6(a) and 6(b)). The results showed significant increases of the tyrosinase activity and melanin content in MCs alkalized by NH_4_Cl or Con A. Furthermore, we also found that intracellular neutralization did not change the expression levels of melanogenic proteins (tyrosinase, MITF, and Pmel17). These results unambiguously demonstrate that the tyrosinase activity of MCs is inhibited by VC and its derivatives through cytoplasmic acidification.

## 4. The Antioxidative Effect of VC Contributes to VC-Mediated Intracellular Acidification via SVCT-2

**Figure 7:**
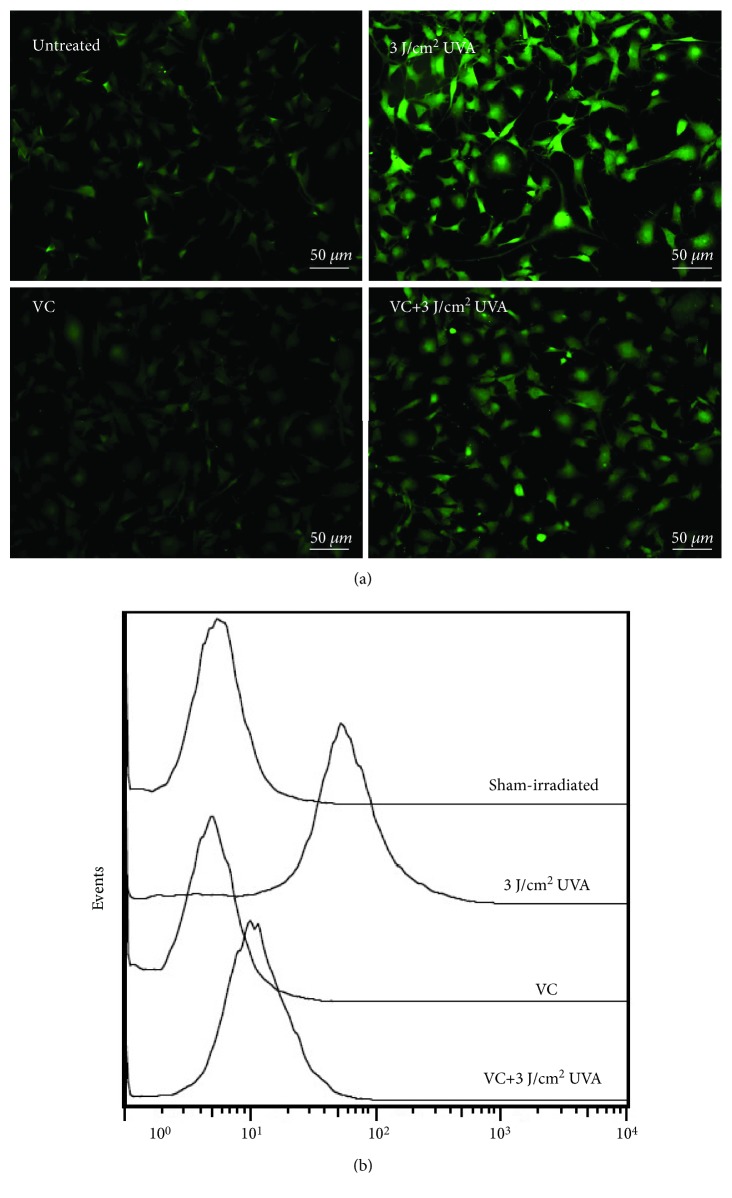
Detection of ROS level by fluorescence microscopy and flow cytometry analysis using DCFH-DA labeling. MCs were exposed to 3 J/cm^2^ UVA for 24 h and were then treated with 1 mM VC for an additional 24 h. Untreated or treated cells were loaded with 10 *μ*M DCFH-DA in serum-free medium for 30 min in the dark. The labeled MCs were observed using a fluorescence microscope (a) and were (b) quantitatively analyzed using a BD FACSCalibur flow cytometer. Scale bar: 50 *μ*m.

**Figure 8:**
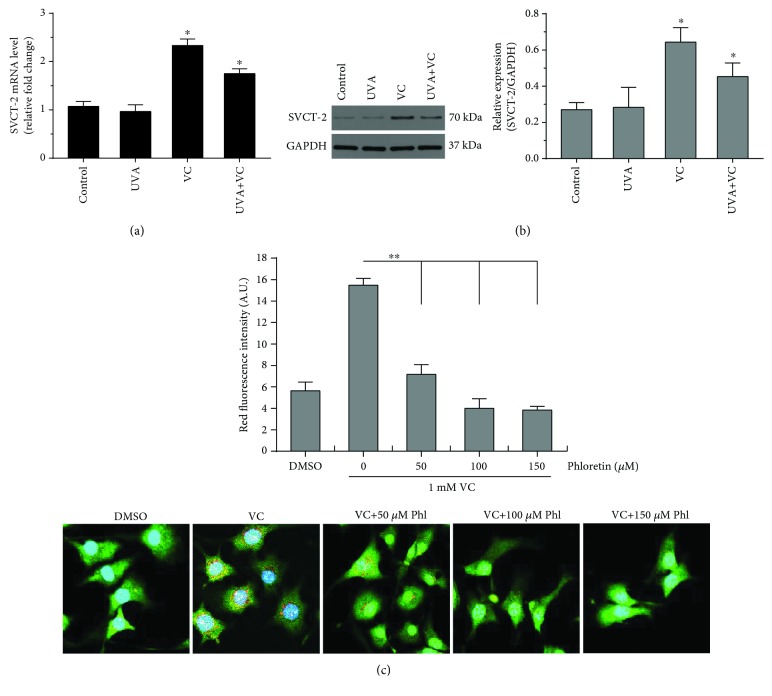
Expression levels of SVCT-2 mRNA and protein in MCs. MCs were seeded in 6-well plates and were then exposed to 3 J/cm^2^ UVA irradiation in the presence or absence of 1 mM VC. (a) The mRNA level of SVCT-2 was measured using qPCR as detailed in Materials and Methods. (b) The protein level of SVCT-2 was analyzed by western blotting using an anti-SVCT-2 antibody. GAPDH was used as a loading control. Representative blots are shown. Data are shown as means ± SD of three independent experiments. ^∗^ *P* < 0.05, compared to the untreated control. (c) For inhibiting SVCT-2, phloretin (a putative SVCT-2 inhibitor) was purchased from Selleck Chemicals (Cat# S2342, Shanghai, China). All compounds were first dissolved in dimethyl sulfoxide (DMSO) and then diluted with PBS into the indicated concentrations. The concentration of phloretin was confirmed to be nontoxic by a cell viability assay. Cells were treated and then stained with AO using the same procedure described in Figure 4. The equal volume of DMSO in PBS was used as a solvent control. Red fluorescence intensity (arbitrary units (A.U.)) for AO staining was measured using ImageJ. Histogram shows the results determined on 50 cells which are presented as means ± SD for three independent experiments. ^∗∗^ *P* < 0.01, versus only VC treatment. Representative immunofluorescence images are shown in the bottom. Scale bar: 10 *μ*m.

To examine whether VC has a scavenging effect against ROS in MCs under oxidative stress, we first measured the intracellular ROS levels of MCs treated with 1 mM VC in the presence or absence of UVA exposure (3 J/cm^2^) using the fluorescent probe DCFH-DA. The intracellular ROS level, indicated by the fluorescence intensity of DCFH-DA, significantly increased in MCs exposed to UVA irradiation, whereas the UVA-induced oxidative stress was attenuated in MCs treated with VC (Figure 7). These data suggest that VC exerts a potent antioxidative potential, which compensates for the potential insufficiency of antioxidant capacity in MCs that results from decreased melanin production. To clarify the mechanism that underlies the effects of intramelanocytic acidification, the expression of two sodium-dependent VC transporter isoforms (SVCT-1 and SVCT-2) was characterized by qPCR and western blotting. The level of SVCT-1 mRNA was virtually undetectable in MCs (data not shown). This observation was inconsistent with previous reports that SVCT1 is primarily found in the epidermis, possibly expressed by keratinocytes [16]. Interestingly, significant increases in the expression of SVCT-2 mRNA and protein were detected in MCs treated with 1 mM VC (Figures 8(a) and 8(b)). In contrast, in the absence of VC treatment, UVA alone did not induce an increase in SVCT-2 expression. To further define the effect of SVCT-2 on intramelanocytic acidification, the cells were cotreated with VC and phloretin. The result indicated that VC-mediated intracellular acidification was neutralized by phloretin in a dose-dependent manner (Figure 8(c)). These results suggest that the enhanced expression of SVCT-2 may provide a rapid transmembrane transport for VC that facilitates intramelanocytic acidification and confers potent antioxidative activity.

## 5. Discussion

Ameliorating hyperpigmentation on the exposed surface of the body and obtaining a lighter skin complexion for both medical and social reasons are greatly valued, especially by Asian females [17]. HQ, a monophenol compound, was first recognized to be a skin lightening ingredient and was widely used for decades as a gold standard among topical remedies for hyperpigmentation in the clinic [1, 18]. However, much recent attention has been paid to the potential health risks of long-term exposure to HQ, such as exogenous ochronosis, leukoderma (occupational vitiligo), and even carcinogenesis [19]. For these reasons, many research groups are currently in pursuit of possible topical alternatives to HQ for skin lightening applications [1].

It is worthwhile to note that the antimelanogenic mechanism employed by most skin lightening agents mediates the suppression of tyrosinase activity at various levels [20, 21]. Although there is little clinical evidence to support the contention that the overuse of skin lightening agents, which are often present in sunscreens and in cosmetic products, may weaken the photoprotective ability of the skin [22], we cannot completely exclude the possibility that the inhibition of melanin synthesis in the skin is probably harmful and increases the genetic damage caused by UV radiation and thus contributes to the continuous rising trend of malignant melanoma [23]. VC has been widely used as a skin lightening agent in cosmetic products for a long time [24, 25]; however, little is known about its mechanism of antimelanogenic action. Herein, we present data showing that VC and its derivatives possess antimelanogenic and antioxidative properties, which may be potential candidates to replace classical tyrosinase inhibitors such as HQ [21].

Our present analyses demonstrate that VC and its derivatives, MAP and AAE, show similar inhibitions of tyrosinase activity and melanin content in a dose-dependent manner, but no changes in the expression levels of tyrosinase, MITF, or Pmel17 proteins in MCs treated with VC, MAP, or AAE were detected by western blotting (Figure 3). Moreover, we further examined changes of intramelanocytic pH using two pH-sensitive fluorescent dyes. The results reveal that significant acidification occurs in MCs treated with VC, MAP, or AAE and the inhibition of tyrosinase activity disappeared following the intracellular neutralization of MCs induced by NH_4_Cl or Con A (Figures 4 [Fig fig5]–6). It has been well established that pH is an essential factor in the regulation of melanogenesis, not only because cytoplasmic pH dramatically affects the catalytic activity of tyrosinase but also because cytoplasmic pH is critical for the assembly of Pmel17 amyloid fibrils that serve as a scaffold in melanosomes upon which melanin is deposited [8, 26, 27]. Previous studies have demonstrated that the maximum melanin yield was obtained at a pH of around 6.8 and was greatly reduced at pH < 5.5 [28]. In addition, we also found that ROS levels were reduced in MCs treated with VC following UVA exposure (Figure 7), which suggests that VC has a scavenging effect against ROS under UVA-mediated oxidative stress. To gain more insight into the regulation regarding the transmembrane transport of VC, we used western blotting analysis and qPCR to assess the protein and mRNA levels of SVCT-2 in MCs treated with VC. As shown in Figures 8(a) and 8(b), there was a significant increase in the expression of SVCT-2 in MCs treated with 1 mM VC. Further, to determine whether SVCT-2 function was associated with intramelanocytic acidification, we analyzed the effect of phloretin (a known SVCT-2 inhibitor) on intracellular pH. The results showed that the intracellular acidification of melanocytes induced by VC was neutralized by phloretin in a dose-dependent manner (Figure 8(c)). This finding provides a convincing evidence that intramelanocytic acidification is likely mediated by VC uptake via SVCT-2.

Our previous study has demonstrated that HQ exerts a direct toxic effect on melanosomal ultrastructure *in vitro* and *in vivo* models [1]. It is noteworthy that HQ presumably acts as a substrate to competitively inhibit tyrosinase, granting the potential of skin lightening. Meanwhile, these monophenol compounds also give rise, through the enzymatic oxidation by tyrosinase, to abundant free radicals that cause lipid peroxidation and consequent melanosomal membrane damages [29]. Therefore, the melanocytic acidification induced by an intracellular pH modulator (such as VC) seems to represent an efficient way to repress tyrosinase activity without direct cytotoxicity against MC *per se*.

In conclusion, this study demonstrates that VC suppresses the tyrosinase activity through cytoplasmic acidification, which is achieved by enhancing the expression of SVCT-2 that potentially facilitates the transmembrane transport of VC. To our knowledge, no previous studies have comprehensively demonstrated that intramelanocytic acidification by VC and its derivatives inhibits the tyrosinase activity, which offers an intriguing mechanism to develop new skin lightening agents to combat undesired hyperpigmentation of the skin.

## Data Availability

The data used to support the findings of this study are available from the corresponding author upon request.

## Supplementary Materials

Supplementary Materials Figure S1: cell viability of melanocytes treated with varying concentrations (0–150 *μ*M) of phloretin or solvent dimethyl sulfoxide (DMSO). Phloretin (a putative SVCT-2 inhibitor) was purchased from Selleck Chemicals (Cat# S2342, Shanghai, China). This compound was first dissolved in DMSO and then diluted with PBS into the indicated concentrations. Cell viability was determined using a cell counting kit-8 reagent, as detailed in Materials and Methods. The equal volume of DMSO in PBS was used as a solvent control. The concentration of phloretin was confirmed to be nontoxic as compared with the solvent control, which was then used for acridine orange fluorescent staining. All data are presented as means ± SD for each treated group compared with the control group from three independent experiments. ^∗^ *P* < 0.05.
