# Supplementary material for: Intramelanocytic Acidification Plays a Role in the Antimelanogenic and Antioxidative Properties of Vitamin C and Its Derivatives
Source: Oxid Med Cell Longev. 2019 May 12;2019:2084805. doi: 10.1155/2019/2084805 (PMC6535878; doi:10.1155/2019/2084805)
Supplement: Supplementary Materials — Figure S1: cell viability of melanocytes treated with varying concentrations (0–150 μM) of phloretin or solvent dimethyl sulfoxide (DMSO). Phloretin (a putative SVCT-2 inhibitor) was purchased from Selleck Chemicals (Cat# S2342, Shanghai, China). This compound was first dissolved in DMSO and then diluted with PBS into the indicated concentrations. Cell viability was determined using a cell counting kit-8 reagent, as detailed in Materials and Methods. The equal volume of DMSO in PBS was used as a solvent control. The concentration of phloretin was confirmed to be nontoxic as compared with the solvent control, which was then used for acridine orange fluorescent staining. All data are presented as means ± SD for each treated group compared with the control group from three independent experiments. ∗ P < 0.05. [file 2084805.f1.docx]

**Supplementary Materials and Methods**

*Determination of cell viability by CCK-8 assay.* The CCK-8 assay kit (Beyotime Biotechnology, Nanjing, China) was used as a qualitative index of cell viability according to the manufacturer’s instructions. Phloretin (a putative SVCT-2 inhibitor) was purchased from Selleck Chemicals (Cat#: S2342, Shanghai, China). In short, MCs were seeded at 5×10^3^ per well in 96-well plates, allowing the cells to attach the bottom of the wells. Phloretin was first dissolved in dimethyl sulfoxide (DMSO) and then diluted with PBS into varying concentrations (0–150 μM). After 48 h, 20 μl CCK-8 was added to each well and incubated at 37℃ for 1 h. The optical density (OD) value was recorded at 450 nm using a microplate reader (Perkin Elmer, Waltham, MA, USA).

**
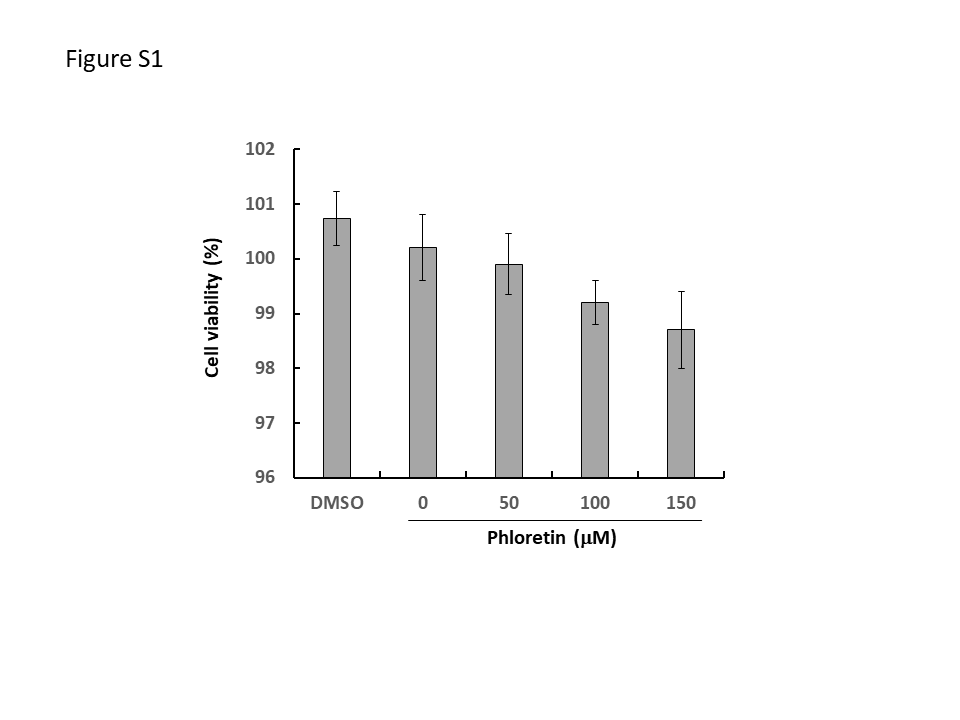
**

Figure S1. Cell viability of MCs treated with varying concentrations (0–150 μM) of phloretin with a group of blank control (only DMSO without cells) and a group of untreated control (cells only treated with medium). Cell viability was determined using the CCK-8 assay kit as described in the Materials and Methods. The concentration of phloretin was confirmed to be non-toxic as compared with the solvent control, which then used for acridine orange fluorescent staining. All data are presented as means ± SD for each treated group compared with the blank control group from three independent experiments. *P <0.05.
